# The association between patient sharing network structure and healthcare costs

**DOI:** 10.1371/journal.pone.0234990

**Published:** 2020-06-22

**Authors:** Kimberley H. Geissler, Benjamin Lubin, Keith M. Marzilli Ericson

**Affiliations:** 1 Department of Health Promotion and Policy, School of Public Health and Health Sciences, University of Massachusetts Amherst, Amherst, MA, United States of America; 2 Information Systems, Boston University Questrom School of Business, Boston, MA, United States of America; 3 Gehr Center for Health Systems Science, Keck School of Medicine of the University of Southern California, Los Angeles, CA, United States of America; Keck School of Medicine of the University of Southern California, UNITED STATES

## Abstract

**Study question:**

While physician relationships (measured through shared patients) are associated with clinical and utilization outcomes, the extent to which this is driven by local or global network characteristics is not well established. The objective of this research is to examine the association between local and global network statistics with total medical spending and utilization.

**Data source:**

Data used are the 2011 Massachusetts All Payer Claims Database.

**Study design:**

The association between network statistics and total medical spending and utilization (using standardized prices) is estimated using multivariate regression analysis controlling for patient demographics and health status.

**Data collection:**

We limit the sample to continuously enrolled commercially insured patients in Massachusetts in 2011.

**Principal findings:**

Mean patient age was 45 years, and 56.3% of patients were female. 73.4% were covered by a health maintenance organization. Average number of visits was 5.43, with average total medical spending of $4,911 and total medical utilization of $4,252. Spending was lower for patients treated by physicians with higher degree (p<0.001), eigenvector centrality (p<0.001), clustering coefficient (p<0.001), and measures reflecting the normalized degree (p<0.001) and eigenvector centrality (p<0.001) of specialists connected to a patient’s PCP. Spending was higher for patients treated by physicians with higher normalized degree, which accounts for physician specialty and patient panel size (p<0.001). Results were similar for utilization outcomes, although magnitudes differed indicating patients may see different priced physicians.

**Conclusions:**

Generally, higher values of network statistics reflecting local connectivity adjusted for physician characteristics are associated with increased costs and utilization, while higher values of network statistics reflecting global connectivity are associated with decreased costs and utilization. As changes in the financing and delivery system advance through policy changes and healthcare consolidation, future research should examine mechanisms through which this structure impacts outcomes and potential policy responses to determine ways to reduce costs while maintaining quality and coordination of care.

**What this study adds:**

- It is unknown whether local and global measures of physician network connectivity associated with spending and utilization for commercially insured patients?
- In this social network analysis, we found generally higher values of network statistics reflecting local connectivity are associated with increased costs and utilization, while higher values of network statistics reflecting global connectivity are associated with decreased costs and utilization.
- Understanding how to influence local and global physician network characteristics may be important for reducing costs while maintaining quality.

## Introduction

Physician relationships measured via shared patients are associated with a large number of clinical and utilization outcomes [1–12]. These relationships, analyzed using social network measures, are a product of formal and informal information sharing [13, 14], organizational affiliations [15, 16], and care coordination [2, 8]. Physician shared patient network structure–based in part on their referral patterns–is impacted by insurance type [17, 18], patient clinical characteristics [19], socioeconomic characteristics [17, 20–22], and organizations [4, 15, 23].

In addition to their association with clinical outcomes, measures of physician patient-sharing network structure have been shown to be associated with spending outcomes [3, 4, 16, 24, 25]. Generally, these studies have found that patients of physicians with less diffuse networks, as measured by local social network measures, have lower spending and utilization [16, 24, 25]. However, there is substantial variation in what is measured in these studies and the results, necessitating a nuanced interpretation of the role of social networks in spending and utilization. For example, Landon and colleagues (2017) found total spending was higher for patients of physicians with more connections to other physicians, adjusted for physician panel size [25]. Barnett and colleagues (2012), using hospital based professional networks, found that more connections per physician was associated with increased spending, while higher “centrality” of primary care physicians measured using the network measure of betweenness centrality, capturing both local and global information sharing, within a hospital network was associated with lower spending [4]. Pollack and colleagues (2012) used a patient-centered measure and found patients treated by sets of physicians who share high numbers of patients had lower spending [3]. Agha and colleagues (2019) found that patients of PCPs who had stronger relationships with fewer specialists have lower spending [16].

These studies–aside from Barnett et al (2012), who used Medicare data to examine only physicians affiliated with hospitals–have focused primarily on local network measures reflecting physicians and their direct connections to other physicians. However, higher-level network characteristics that describe a physician’s overall position in an information sharing network (e.g. eigenvector centrality), may also be important and may be associated with utilization and spending outcomes.

The identification of quantitative measures of the structural importance of a given physician (node) within a network has been extensively studied in network science. A number of such “centrality” measures, or how involved a given node is in the overall flow of the network, are commonly used, with a long literature related to the applications of these measures [7, 11, 26, 27]. For example, the centrality of participants in a social network has been shown to be important in mediating what information is received from peers, and consequently what choices participants make [28].

Despite a long literature in social network measures, interpreting the impacts of the centrality of nodes within in social networks–and particularly those created somewhat indirectly, through shared-patient patterns–is not necessarily straightforward. On the one hand, central nodes may be exposed to better and more comprehensive information, leading to higher performance. On the other hand, such nodes may be buffeted by too much information, leading to overload and poor performance. The literature on organizational structure and information processing [29, 30] has shown that the overload effect is mitigated in settings where information is well-codified and where topic changes are rare [31], which is likely to be the case in healthcare. Additionally, the centrality of nodes within networks can be measured in several different ways, including with both local connectivity–as described in much of the literature above–and with global centrality in the network, which may contribute differently to spending and utilization patterns. Given differences in the productivity implications of these centrality measures, it is unknown how local and global social network measures are associated with utilization and spending patterns in healthcare.

To fill this gap, and elucidate how the position of a physician within a patient-sharing network is associated with outcomes, we use the physician patient-sharing network of commercially insured patients in Massachusetts to examine the association of both local and global network statistics with patient utilization and costs. In line with previous findings [4, 16, 25], we hypothesize that increased local connectivity will be associated with increased costs and utilization while increased global centrality will be associated with reduced costs and utilization. As changes in the insurance and delivery system impact physician referral patterns and care coordination efforts, quantifying the impacts of physician interactions at the local and global level are important to understand associations with spending outcomes.

## Methods

### Overview

Using health insurance enrollment and claims data, we construct a patient-sharing network for physicians in Massachusetts caring for commercially insured patients with the two most common insurance types. We calculate a range of statistics summarizing the structure of this network and the local and global connectedness of the physicians within the network.^18^ We then use these statistics to examine the association between these network statistics and patient utilization and cost outcomes.

### Data and analytic sample

Data are from the Massachusetts All Payer Claims Database, version 1.0 [32], and include health insurance enrollment and claims data (inpatient and outpatient) for commercially insured enrollees aged 21–64 in 2011. This includes insurance from employer-sponsored insurance, self-insured employers, health insurance marketplaces, and individually purchased plans. We use all commercially insured enrollees to construct the patient-sharing network as described below. For the analytic sample used to analyze utilization and cost outcomes, we limit to enrollees who were continuously insured for all of 2011 in a health maintenance organization (HMO) or preferred provider organization (PPO) plan who have at least one face-to-face visit with a physician included in the network.

#### Construction of patient-sharing network and network statistics

We construct a physician patient-sharing network using links between two physicians identified by patients they have in common over a one-year period [1]. We include “face-to-face” visits, defined as evaluation and management visits or procedures with a relative value unit greater than two [1]. Following the previous literature, we limit links between physicians to those who share at least 9 patients [1, 33], which has been shown to reflect a meaningful clinical relationship among physicians in an academic medical center [13]. We limit to PCPs, medical specialists, and surgical specialists with a modal ZIP code in Massachusetts. The network is constructed using weights reflecting the number of patients shared between physicians.

We calculate a number of network statistics reflecting the local and global connectivity of physicians in the network (see S1 Appendix). The local measures we use are *degree*, *normalized degree*, *and clustering coefficient*. *Degree* is the number of connections incident to a given physician, and is heavily influenced by patient panel size. *Normalized degree* is a measure that captures the local connectivity of physicians relative to their patient panel size and specialty [34]. Consistent with the literature [1], normalized degree adjusts for patient panel size by using regression instead of simple division. Further, it includes an additional adjustment for physician specialty as panel size and patient-sharing patterns vary substantially between PCPs and specialists [34]. Given these differences, we anticipate there may be different findings between degree and normalized degree. *Clustering coefficient* is a measure of network density, reflecting the embeddedness of a physician within a local cluster; clustering coefficient is the empirical probability that physicians adjacent to a given physician are connected to one another and therefore has a built-in accommodation for panel size in its calculation. For example, Dr. Jones shares patients with Dr. Hernandez and with Dr. Smith; the clustering coefficient reflects the probability Dr. Hernandez and Dr. Smith share patients with each other. Clustering coefficient is commonly reported in the network science literature, and is interpreted as the density of local connectivity. The global measure we use is *eigenvector centrality*, which is a measure of global information flow where high values occur for those physicians who are not only highly connected, but are also connected to other physicians who are highly connected, and so on. Due to its interpretation as the probability a random walk over the network will land on a given node (physician), eigenvector centrality can be interpreted as reflecting “important” physicians in the sense of global information flow over the network.

**Fig 1:**
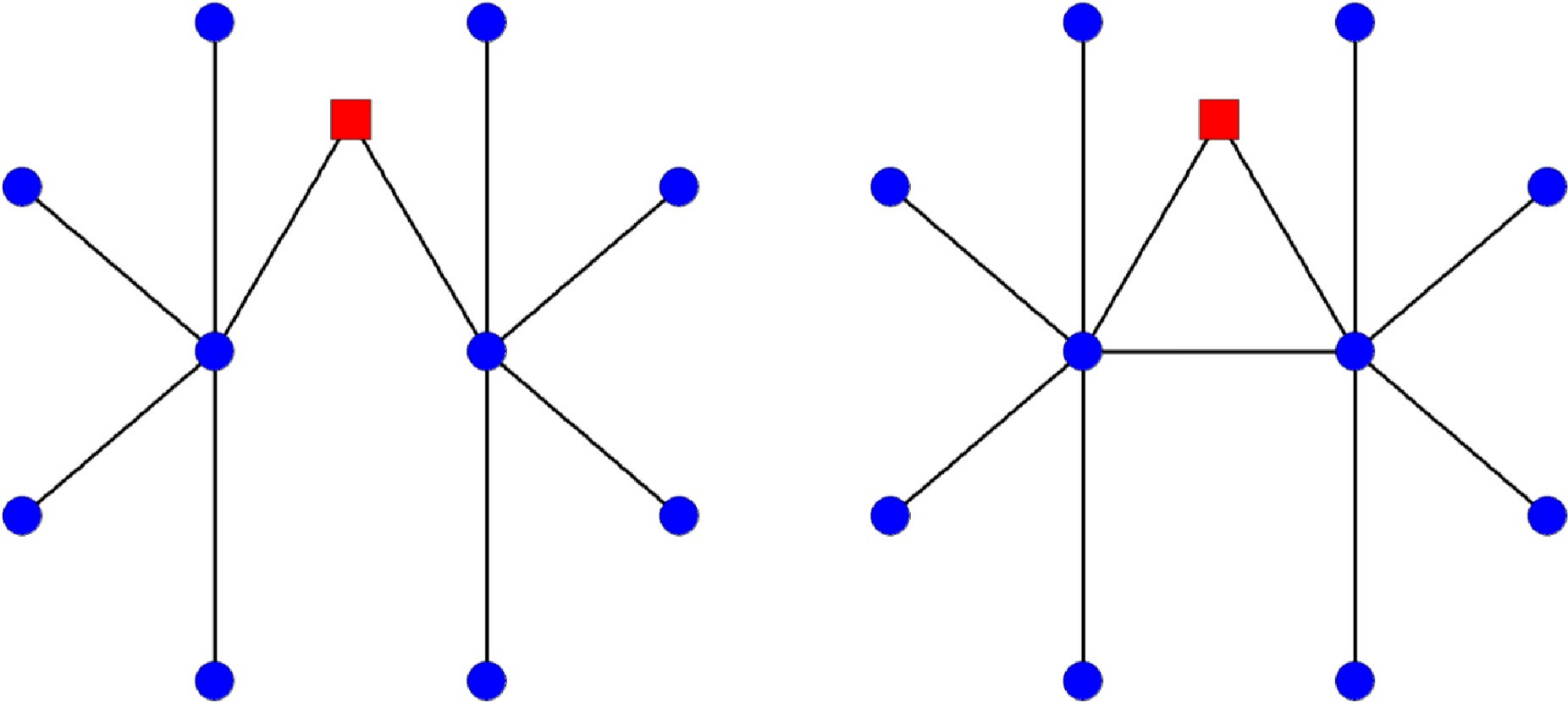
Diagram of example network to show differences in local and global connectivity measures.

Fig 1 provides an example of the difference in local and global connectivity by diagramming two networks that differ by the presence of a single link. Without the link (left), the square red node has a high eigenvector centrality and a degree of two. With the added link (right), there is no change in the degree of the square red node, but the node’s eigenvector centrality has been reduced because it is more easily bypassed in a random walk of the network–it has become less important. This illustrates that adding links can decrease centrality, showing that the relationship between links and global centrality measures is complex.

We additionally examine network statistics of specialists connected to a patient’s PCP using two measures of *referral centrality*: *normalized degree referral centrality* and *eigenvector referral centrality*. These statistics [34] reflect the local and global centrality of the average of network statistics of specialists sharing patients with a given PCP, weighted by the number of patients they have in common. Thus, these statistics are only available for PCPs, and reflect the network structure of specialists with whom they work.

We examine network statistics at the patient level. We assign network statistics to patients using a visit-weighted average of network statistics for all physicians seen by a patient; this assigns the network statistics to patients proportionally to the number of visits over the course of the year. For the referral centrality measures, which are calculated for PCPs only, we again use this visit-weighted method (although most patients see only one PCP).

### Statistical analysis

The primary outcomes of interest are patient level total medical spending and total medical utilization for the full year of 2011. These measures include all inpatient and outpatient spending, but do not include pharmaceutical spending. We calculated total medical spending as the final allowed amount for each visit/procedure, including all insurer and patient paid amounts. As these negotiated allowed amounts vary substantially across insurers and physicians [35, 36], we calculate standardize prices for each procedure observed. These standardized prices were calculated as the mean price at the procedure-procedure modifier level and account for the quantity of services provided. These standardized prices are a dollar-weighted measure of utilization, hence we refer to this as total medical utilization.

We calculate descriptive statistics for patient characteristics and the patient level network statistics. To estimate the association between network characteristics and spending, we use multivariate linear regression analysis for the dependent variables of logged patient level total medical spending and logged total medical utilization. Due to the right skewed distribution of patient spending, and the fact that all patients have some spending due to the limitation to at least one visit during the year, we use the natural logarithm of spending measures as the outcomes. The primary independent variables are the patient visit-weighted network statistics previously described. We include additional controls in the model, specifically insurance type (i.e., HMO vs. PPO), insurer, age (and age-squared), sex, an interaction of age and sex, and indicator variables for patient 3-digit ZIP code. We also included a set of 162 indicator variables of hierarchical condition categories (HCC) calculated using the Massachusetts Hierarchical Condition Categories algorithm [37, 38]. These include indicators of conditions such as diabetes with acute complications, depression, and heart failure. We used robust standard errors in all regressions to correct for heteroskedasticity [39].

Data management and statistical analysis were conducted using SAS 9.3 (Cary, NC) and Stata-MP 12.1 (College Station, TX). Network analyses were implemented using Python and the igraph package in R [40]. An alpha of 0.05 was considered statistically significant. The study was reviewed and approved by the Boston University Institutional Review Board.

## Results

**Fig 2:**
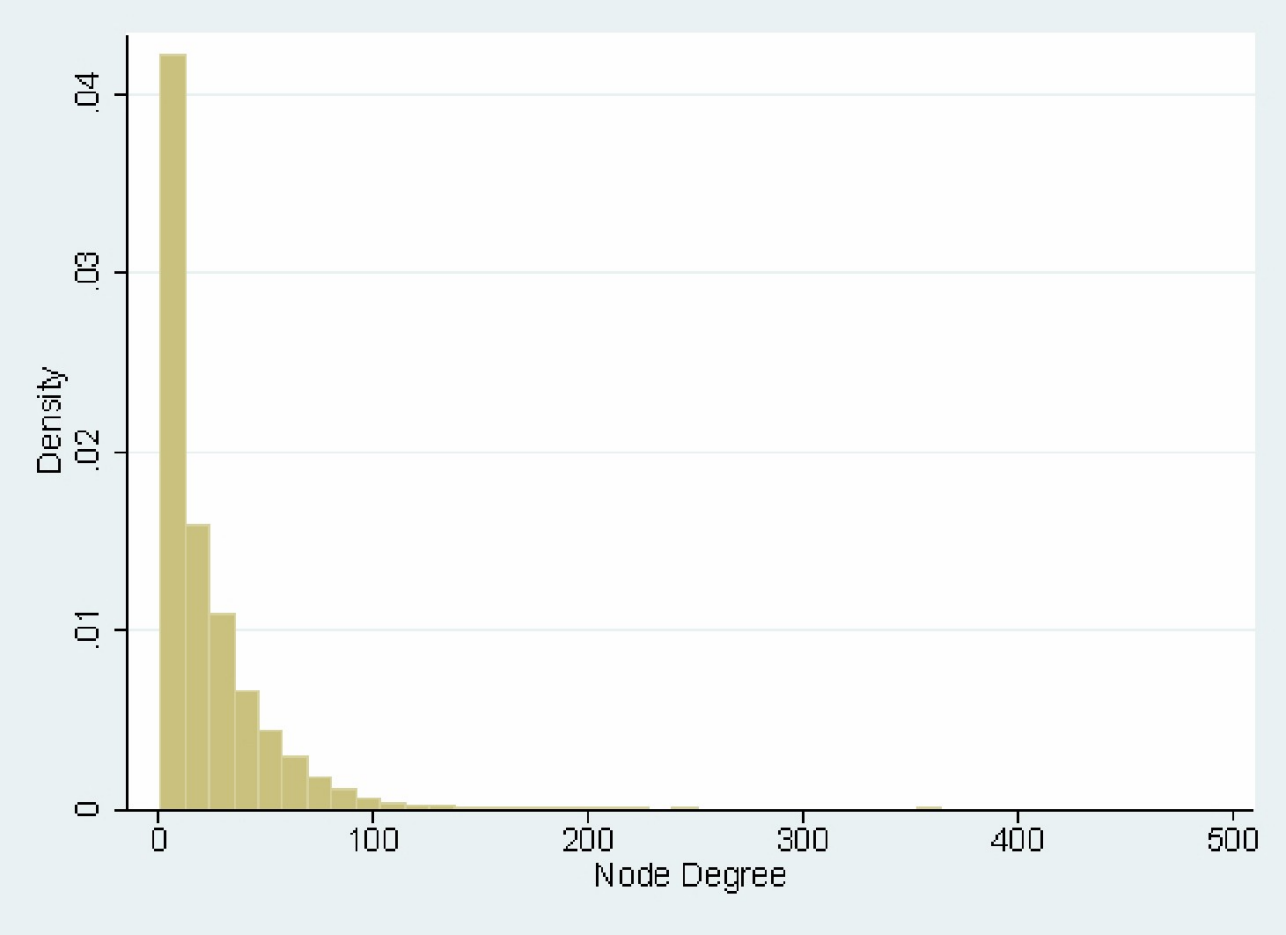
Distribution of degree for physicians included in the patient-sharing network. N = 11,693 physicians. Physician level statistic including physicians in the main patient-sharing network; links with other physicians of at least 9 patients are included.

We include 984,479 patients seen by 11,639 physicians in Massachusetts with calculated network statistics. Of these physicians, 36% are PCPs, 53% are medical specialists, and 11% are surgical specialists. Average patient panel size (number of unique patients for face-to-face visits) is 386, with larger patient panels for PCPs than medical or surgical specialists. The network is highly connected; the degree distribution is shown in Fig 2, which shows (for those who share at least 9 patients) the number of shared patients with other physicians in the network at the physician level. Patient-level descriptive statistics (Table 1) show that, for patients with at least one face-to-face visit, average spending was $4,911 per patient. Weighted average network statistics are shown as well.

**Table 1:** Patient-level descriptive statistics.

	Mean (standard deviation) or %
*Patient characteristics*	
HCC Risk Score	1.34 (3.07)
Age in years	45.04 (11.53)
Female	56.3%
Insurance Type is HMO (versus PPO)	73.4%
Face-to-face Visits (2011)	5.43 (6.54)
Total Medical Spending (2011)	$4911 (16536)
Total Medical Utilization (2011)	$4252 (13994)
*Network statistics (visit-weighted)*	
Degree (local)	40.82 (32.77)
Normalized Degree (local)	20.27 (16.02)
Clustering Coefficient † (local)	0.5169 (0.1692)
Eigenvector Centrality (global)	0.0171 (0.0598)
Normalized Degree Referral Centrality‡ (local)	32.81 (16.67)
Eigenvector Referral Centrality ‡ (global)	0.0244 (0.0638)
Number of patients	984,470

† Over 978,557 patients

‡ Over 759,149 patients.

HCC Risk Scores are a summary measure of health status based on the 162 HCC indicators included in the regression analysis. Here, they are calculated assuming both insurance types are in the “gold” metal tier.

Regression results of the association between network statistics and total medical spending (Table 2), adjusted for patient characteristics, show that patients seeing physicians who are highly locally connected are associated with lower spending for degree and higher spending for normalized degree. The association between degree and lower spending is of very small magnitude, although it is statistically significant. Higher values of other network statistics, including clustering coefficient, eigenvector centrality, and the referral centrality measures, are associated with lower spending.

**Table 2:** Regression adjusted results for association of total medical spending and utilization with network statistics.

	Log of Total Medical Spending	Log of Total Medical Utilization	Number of Observations
Weighted Average			
Degree (local)	-0.00082***	-0.00044***	984,470
	(0.000031)	(0.00003)	
Normalized Degree (local)	0.0022***	0.0043***	984,470
	(0.000066)	(0.000068)	
Clustering Coefficient (local)	-0.516***	-0.460***	978,557
	(0.006)	(0.006)	
Eigenvector Centrality (global)	-0.669***	-0.671***	984,470
	(0.016)	(0.017)	
Normalized Degree Referral Centrality (local)	-0.0047***	-0.0011***	759,149
	(0.000074)	(0.00007)	
Eigenvector Referral Centrality (global)	-0.672***	-0.701***	759,149
	(0.018)	(0.019)	

*: p <0.05

**: p <0.01

***: p <0.001.

Each row represents a separate regression with total medical spending (left column) or total medical utilization (right column) as the dependent variable and the specific network statistic as the primary independent variable. Controls are included for insurance type (health maintenance organization vs. preferred provider organization), age, age-squared, sex, an interaction of age and sex, indicator variables for 162 Hierarchical Condition Categories, indicator variables for insurer, and indicator variables for patient 3-digit ZIP code. Robust standard errors in parentheses.

**Fig 3:**
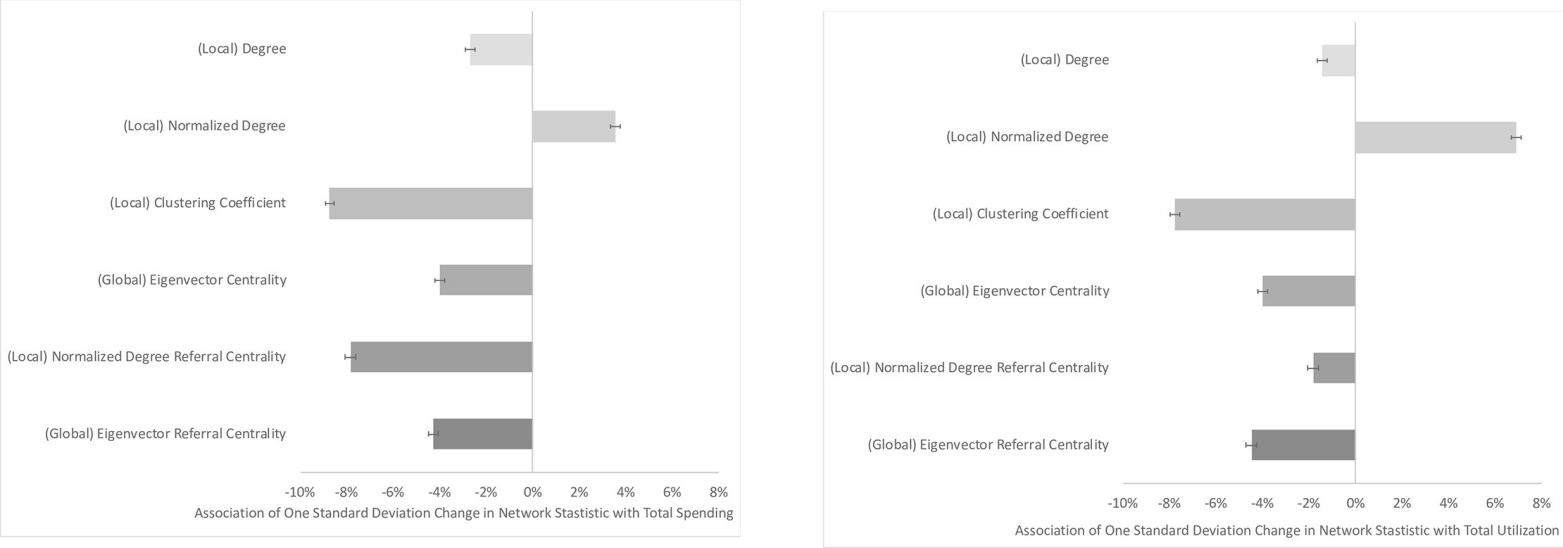
Associations of network statistics with spending and utilization. **Panel A:** Change in total medical spending associated with changes in network statistics. Each row represents the change in total medical spending associated with a one standard deviation change in the network statistic, calculated from a separate regression for each statistic. 95% confidence intervals for the estimates are shown with horizontal bars. Controls are included for insurance type (health maintenance organization vs. preferred provider organization), age, age-squared, sex, an interaction of age and sex, indicator variables for 162 Hierarchical Condition Categories, indicator variables for insurer, and indicator variables for patient 3-digit ZIP code. **Panel B:** Change in total medical utilization associated with changes in network statistics. Each row represents the change in total medical utilization (standardized spending) associated with a one standard deviation change in the network statistic, calculated from a separate regression for each statistic. 95% confidence intervals for the estimates are shown with horizontal bars. Controls are included for insurance type (health maintenance organization vs. preferred provider organization), age, age-squared, sex, an interaction of age and sex, indicator variables for 162 Hierarchical Condition Categories, indicator variables for insurer, and indicator variables for patient 3-digit ZIP code.

Regression adjusted results of the association between network statistics and total medical utilization (using standardized spending) show that, controlling for patient characteristics, patients seeing physicians who are highly locally connected are associated with increased utilization (Table 2). Similar to the results for total spending, higher values of other network statistics, including clustering coefficient, eigenvector centrality, and both referral centrality measures, are associated with lower utilization. The size of these effects is meaningful (Fig 3): a one standard deviation increase in normalized degree is associated with a 6.9% increase in utilization; a one standard deviation increase in eigenvector centrality is associated with a 4.0% decrease in utilization; a one standard deviation increase in clustering coefficient is associated with a 7.8% decrease in utilization; a one standard deviation increase in normalized degree referral centrality is associated with a 1.8% decrease in utilization; and a one standard deviation increase in eigenvector referral centrality is associated with a 4.5% decrease in utilization.

Differences between the results for total spending and total utilization–which differ in that total utilization uses standardized prices across physicians–show that the direction and magnitude of associations is very similar for clustering coefficient and eigenvector centrality (both standard and referral centrality), while it varies in magnitude for the local measures of degree and normalized degree (both standard and referral centrality). This suggests that local connections to higher cost PCPs, and in the case of referral centrality, higher cost specialists, could be driving these differences. For global network statistics, the associations are similar when considering spending and utilization.

## Discussion

We analyze the association of network statistics with medical spending and utilization and find that generally, higher values of network statistics reflecting local connectivity adjusted for physician characteristics are associated with increased costs and utilization, while higher values of network statistics reflecting global connectivity are associated with decreased costs and utilization. These differences are similar for the network as a whole and when restricted to measures of referral centrality, which is based only on the network characteristics of specialists connected to PCPs.

The use of commercial insurance data is particularly important, as it allows us to see differences in spending and utilization, separating differences in utilization from those of using higher or lower priced physicians. Although there is prior evidence that patients treated by diffuse sets of physicians, as measured by network analysis, have higher costs [2, 16, 24, 25], our results are novel in that they carefully examine different ways of understanding the structure of the network and in differentiating local from global network centrality measures. Additionally, understanding the differential role of price from utilization in total medical spending is important for understanding potential mechanisms behind the associations we observe.

These results have similarities and differences to previous work that shows that local measures of connectivity, particularly for PCPs, are positively associated with utilization and spending in both Medicare fee-for-service and commercial insurance [2, 16, 25]. There are potential tradeoffs in the clinical and cost implications of these local network statistics. On the one hand, locally central physicians may be exposed to better and more comprehensive information, leading to higher performance or a better physician-patient match [16]. On the other hand, physicians with a high degree or normalized degree may be less careful with the decisions to initiate referrals and directing referrals to a smaller number of physicians, which would allow them to better coordinate and manage the set of specialists [16, 41].

Our results showing patients treated by physicians with higher global centrality have reduced spending and utilization is consistent with physicians that are more central in the network potentially obtaining improved information from others in the network, and thus effectively mediating physician treatment choice [28]. Understanding high versus low cost referral strategies, both for utilization and prices, and the effectiveness of a given network structure for future healthcare spending is an important contribution with ongoing changes in the delivery system and financing structure related to physician incentives [42, 43]. However, it is possible that optimizing network structure from a cost perspective may be in conflict for a given patient–care must be taken that optimizations made for the long-run efficiency of the system do not compromise patient care.

Unlike local measures of connectivity that could potentially be influenced by relatively straightforward measures such as providing specific lists of specialists for referrals, or encouraging PCPs to limit the number of specialists with whom they work, understanding how to influence the eigenvector centrality of a given physician is substantially more complex. Since this statistic depends not only on the physician’s own actions, but also those of their peers and their peers’ peers, etc. [44], determining concrete actions or changes that will directly influence this is difficult. Future research should examine what types of doctors have higher and lower global centrality measures, and potentially use this as a target within a network of physicians for ensuring that information and clinical practice guidelines are being properly disseminated to all members of the network. For example, prior research has shown that physicians–and particularly PCPs–who treat larger proportion of Medicaid patients have lower eigenvector centrality [17]. Central physicians within the patient-sharing network may be important targets to ensure information diffusion is occurring to optimize patient quality of care at the lowest cost.

There are several limitations of the analysis. The first is that our sample is limited to commercial patients in Massachusetts, and therefore may be different than results in other geographic regions or states. We note that the state has a relatively interconnected healthcare system due to its size and concentration of physicians in the greater Boston area, and so the use of a statewide network captures most healthcare interactions for this patient population. The addition of information about a diverse set of commercial patients for whom we can observe a large proportion of the network [8, 16] is an important contribution to the literature, which is often limited to older Medicare fee-for-service patients or a single commercial insurer [1, 8]. Second, we are not able to separate mechanisms by which local and global centrality measures are associated with utilization and spending. We observe similar impacts for statistics related to both all physicians and those restricted to specialists connected to PCPs, suggesting associations between network structure and costs are due to a mechanism that impacts both specialists and PCPs. Third, there is variation in the literature as to the threshold at which physicians recognize patient sharing relationships and the stability of these relationships over time [45]. The commonly used threshold of nine patients was established through data collection in an academic medical center using Medicare data [13]. Other work has used Medicare data among community-based physicians, physician assistants, and nurse practitioners and found higher thresholds [46]. As similar samples of Massachusetts physicians are primarily based in the community [17], but are also defined using commercial insurance claims from multiple payers covering a larger proportion of the population than Medicare, we use the most commonly used threshold of nine patients such that our work is comparable to other literature [1, 4, 25, 42].

As network science advances in health services research [24], and we learn more about the structure of physician patient-sharing networks, understanding implications of network structure for spending and health outcomes is important. We find nuanced results of associations between local and global network connectivity statistics that suggest there is not a simple path to reducing spending through altering the structure of the physician network. As changes in the financing and delivery system advance through policy changes [42, 43, 47, 48] and healthcare consolidation [35], future research should examine mechanisms through which this structure impacts outcomes and potential policy responses to determine ways to reduce costs while maintaining quality and coordination of care.

## Supporting information

S1 Appendix Definitions of network measures. (DOCX)
