# Supplementary material for: The association between patient sharing network structure and healthcare costs
Source: PLoS One. 2020 Jun 22;15(6):e0234990. doi: 10.1371/journal.pone.0234990 (PMC7307780; doi:10.1371/journal.pone.0234990)
Supplement: S1 Appendix — (DOCX) [file pone.0234990.s001.docx]

**Supplemental Technical Appendix:** Definitions of network measures

*Definitions:* A network is defined by *nodes* (i.e., physicians) and *edges*, which are the links between them. For the network we construct, an edge exists between two physicians if and only if the physicians share at least 9 patients for face-to-face visits. Face-to-face visits are defined as evaluation and management visits or procedures with a relative value unit greater than two.

*Degree:*

For each physician (node), the number of edges incident to them.

*Normalized Degree:*

Our *normalized degree* measure is the degree of a given physician above or below that predicted based on panel size and type. We begin by creating a simple regression of degree as:

$degree= \beta_{0}+\beta_{1}\chi_{Med}+\beta_{2}\chi_{Surg}+\beta_{3}p+\beta_{4}p\chi_{Med}+\beta_{5}p\chi_{Surg}+\epsilon$ (Eqn 1)

Where $\chi_{\mathrm{Med}}$, $\chi_{\mathrm{Surg}}$are indicators for medical and surgical specialists respectively,$p$ is the physician’s panel size, the regression coefficients are given by $\beta_{i}$ and the residual is $\epsilon$.

The mean standard degree of each physician type under the model is:

$\bar{{degree}_{PCP}}=\beta_{0}+\beta_{3}\bar{p_{PCP}}$ For PCPs (2)

$\bar{{degree}_{Med}}=\beta_{0}+\beta_{1}+{(\beta}_{3}+\beta_{4})\bar{p_{Med}}$ For Medical Specialists (3)

$\bar{{degree}_{Surg}}=\beta_{0}+\beta_{2}+{(\beta}_{3}+\beta_{5})\bar{p_{Surg}}$ For Surgical Specialists (4)

Where $\bar{p_{PCP}}$, $\bar{p_{Med}}$, and $\bar{p_{Surg}}$ are the mean panel sizes for PCP, Medical Specialists, and Surgical Specialists, respectively. Our new *normalized degree* measure for a physician $i$ is then defined as:

${normalized degree}_{i}=\left\{ \begin{aligned} \bar{{degree}_{PCP}}+\epsilon_{i} \\ \bar{{degree}_{Med}}+\epsilon_{i} \\ \bar{{degree}_{Surg}}+\epsilon_{i} \end{aligned} \right.$

For PCPs

For Medical Specialists (5)

For Surgical Specialists

The measure will not be mean zero by design, as a physician of mean panel size for their specialty will retain their true degree for the normalized degree.

*Clustering Coefficient:*

The clustering coefficient for a certain physician (node) $i$ is defined by considering its neighborhood, $N_{i}$*,* which is the set of all physicians to which $i$ is connected by edges. The *clustering coefficient* is given by the total number of edges among physicians in the neighborhood$N_{i}$, divided by the total possible number of such connections. Specifically, this divisor is given by *(*$|N_{i}|\cdot|N_{i}-1|$*)/*2.

*Eigenvector Centrality:*

The *eigenvector centrality* for a certain physician $i$ is calculated as the $i^{th}$ component of the non-negative eigenvector of the adjacency matrix representing the network (we calculate it using R’s igraph package). One interpretation of the measure is as the probability that a sufficiently long random walk over the network will land on physician $i$. Eigenvector centrality is a common way to measure influence in a network (e.g., Google’s PageRank algorithm is similar). As a measure of influence, eigenvector centrality measures not only the strength of the ties incident to $i$, but the strength of ties incident to physicians connected to $i$, and the strength of ties incident to those physicians, and so on. As such, it is a *global* measure of influence.

*Referral Centrality Measures (Normalized Degree and Eigenvector Centrality)*

For each of these measures, available only for PCPs, we take the previously calculated network measures as described above for medical and surgical specialists and assign them to PCPs using a weighted average. The weight used in the weighted average is the number of patients each PCP shares with each specialist.
